# Complete chloroplast genome of *Zingiber mioga* by *de novo* sequencing

**DOI:** 10.1080/23802359.2021.1904799

**Published:** 2021-03-26

**Authors:** Ping Jiang, Renshu Huang, Taotao Sun, Cunwu Chen, Ruihua Zuo, Ying Taoa

**Affiliations:** a College of Biological and Pharmaceutical Engineering, West Anhui University, Lu’an, PR China; b Anhui Engineering Laboratory for Conservation and Sustainable Utilization of Traditional Chinese Medicine Resources, Lu’an, PR China

**Keywords:** *Zingiber mioga*, phylogenetic tree, chloroplast genome

## Abstract

*Zingiber mioga (Thunb.) Rosc.* (*Zingiber mioga*) is an important edible species, which also has important medical and natural pigment value. This article is firstly reported the *Zingiber mioga’s* chloroplast genomes which detect by de novo sequencing. The results showed that the length sequence of *Zingiber mioga’s* chloroplast genome was 163,541 bp, and the length of LSC, SSC, and two IR regions was 88,035, 15,886, and 29,810 bp, respectively. *Zingiber mioga’s* chloroplast genome was encoded 135 genes involving 10 *rRNA*, 38 *tRNA*, and 87 protein-coding genes. After phylogenetic and cluster analysis, the *Zingiber* were closest approach to *Zingiber mioga*, followed by *Kaempferia*, *Curcuma*, *Hedychium*, and *Roscoea*.

*Zingiber mioga (Thunb.) Rosc.* (*Zingiber mioga*) is a Zingiberaceae family from China, Japan, and Korea. Its young flower buds and fruit has been used as a traditional food (Jo et al. 2016), and it is used medicinally to treat the irregular menstruation, dysmenorrhea, cough, and asthma in China and consumed throughout Japan (Huang et al. 2016; Lee et al. 2016;. Moreover, *Zingiber mioga* also has important medical and natural pigment value (Huang and Li 2013; Huang et al. 2016). However, due to human over-exploitation and consumption of wild resources, the *Zingiber mioga* resources have been faced seriously challenges in China. Compared with nuclear genome, the chloroplast genome has conserved structure and orthologous (Aldrich et al. 1988; Yi et al. 2016; Ali et al. 2018; Wang et al. 2020), and it plays an important role in *Zingiber mioga’*s heredity and evolution. Nonetheless, there are no related studies about the *Zingiber mioga’*s chloroplast genome, that blocked the process of molecular genetics research. Therefore, we submit a complete *Zingiber mioga’*s chloroplast genomes by *de novo* sequencing.

We collected the fresh leaves of *Zingiber mioga* (stored in herbarium of Traditional Chinese Veterinary Medicine Laboratory of West Anhui University, Voucher numbers: TCVM202004150125) from Yuexi county, Anhui province, PR China (N:30.84939°, E:116.35999°), and used Plant DNA extraction kit (TIANGEN, Beijing, China) to extract total DNA of *Zingiber mioga’*s fresh leaves, and then we use micro-volume spectrophotometer (OD_260 nm_ and OD_280 nm_) and 1% agarose electrophoresis method to detect DNA quality. When the DNA quality meets the sequencing requirements, the DNA was sent to Beijing Zhongxing Bomai Technology Co., LTD for sequencing using Collibri PCR-free PS DNA Library Prep Kit for Illumina Systems by Illumina NovaSep platform (template size: 500 bp). The Raw data were filtered to obtain Clean Data, then the Get Organelle pipeline (https://github.com/Kinggerm/GetOrganelle) was been ran to cut out the top 15 million reads from Cleandata, and then the SOAP de novo software (Luo et al. 2012) was used to assemble the Clean data to obtain the contig sequence. The BLAT (Kent 2002) was used to get the relative position of the genome in the contig sequences reference to the genome (NC 024157.1, NC 011942.1, NC 009618.1, NC 000932.1, and KX 352464.1). The Bandage tool (Wick et al. 2015) was run to obtain the full-length frame diagram of chloroplast genome. The Geseq program (https://chlorobox.mpimp-golm.mpg.de/geseq.html) was used to annotate chloroplast genome. The OGDRAW software (Lohse et al. 2013) was used to draw the physical map of chloroplast genome (GenBank accession number: MW285081).

The result of genome analysis showed that the *Zingiber mioga’*s chloroplast genome has a typical four-segment structure, including a large single copy region (LSC), a small single copy region (SSC), and two inverted repeat region (IR) (Figure 1). And the full-length sequence of *Zingiber mioga’*s chloroplast genome was 163,541 bp, with the length 88,035 bp, 15,886 bp, and 29,810 bp of LSC, SSC, and two IR regions, respectively. Its GC content was 36.04% and encoded 135 genes involving 10 *rRNA*, 38 *tRNA*, and 87 protein-coding genes.

**Figure 1.**
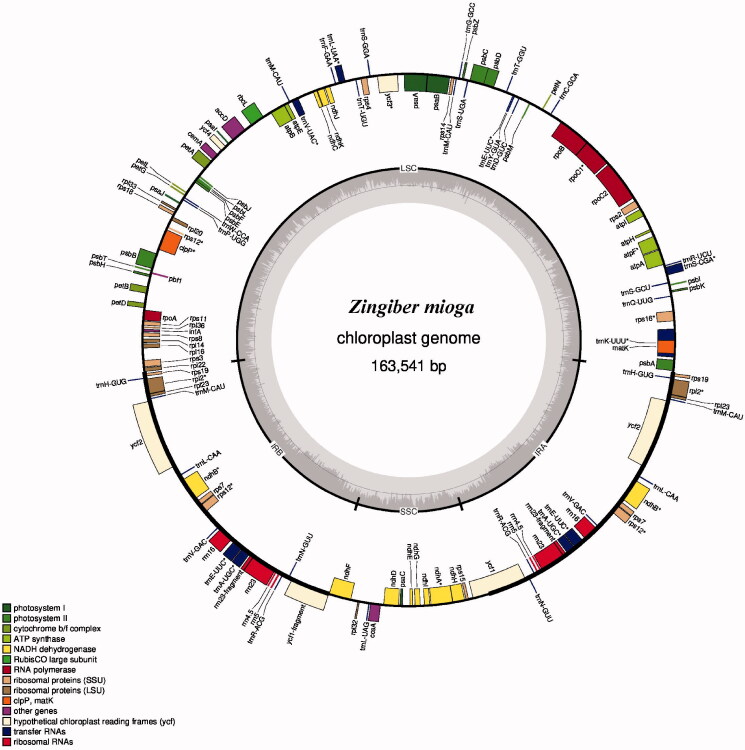
Gene map of the *Zingiber mioga*’s chloroplast genome.

To confirm the phylogenetic status of *Zingiber mioga* in *Scitamineae* plants, we used 87 chloroplast protein-coding genes from eight *Zingiberales* plants and 1 out group (*Ravenala*) in NCBI for phylogenetic analysis and performed raxmlGUI version 1.5 b (https://sourceforge.net/projects/raxmlgui/) by GTRCATX model with 1000 bootstrap replicates. All 10 plants were divided into seven genus. The first genus consisted of two *Zingiber*, which showed that *Zingiber spectabile* and *Zingiber mioga* have a close relationship. The second genus was consisted by *Kaempferia galanga* and *Kaempferia elegans*, and the third and fourth genus were consisted by *Curcuma*, *Hedychium* and *Roscoea,* that four genera has a close relationship. However, *Wurfbainia longiligularis* was far apart from other *Zingiberales* plants. And *Ravenala madagascanensis* (*Musaceae*) as outgroup was away from each other plants. The cluster analysis results showed that *Zingiber* were closest to *Zingiber mioga*, followed by *Kaempferia*, *Curcuma*, *Hedychium*, and *Roscoea* (Figure 2). This research provides a basis for molecular genetics research for *Zingiber mioga*’s classification.

**Figure 2.**
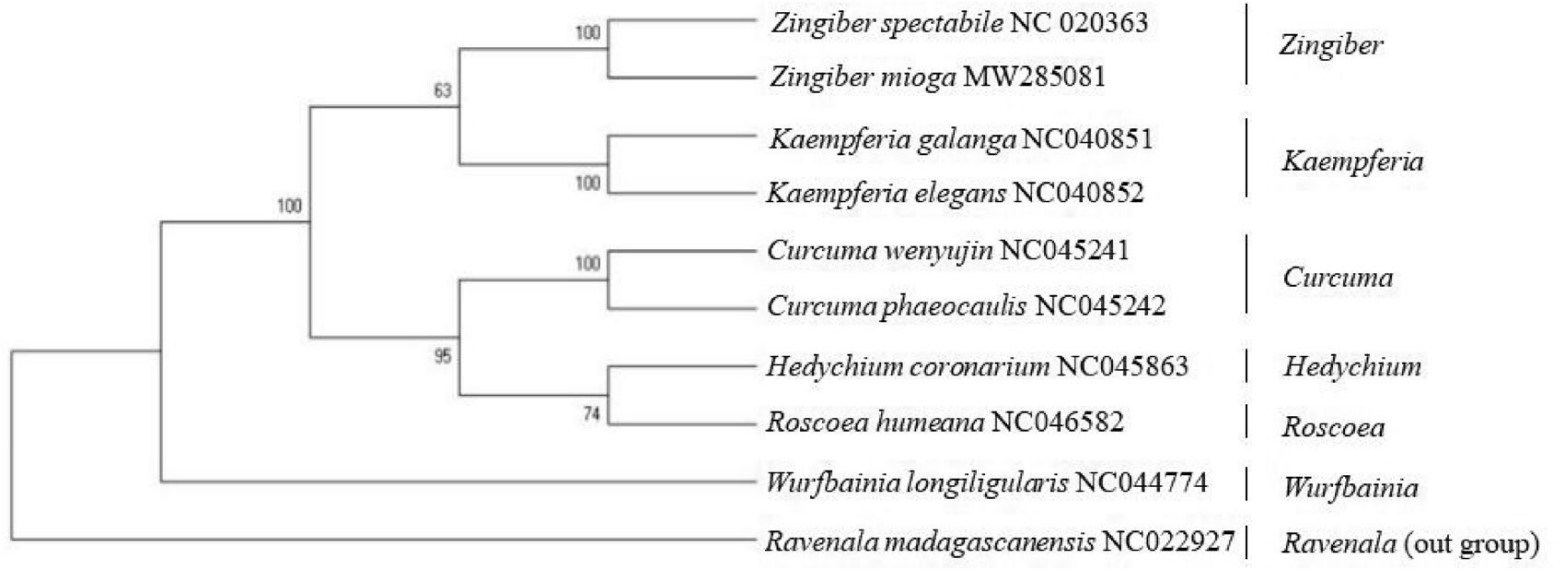
The ML phylogenetic tree of the *Zingiber mioga*.

## Data Availability

The data that support the findings of this study have sent it up to BankIt (2403323) of National Center for Biotechnology Information, and provided GenBank accession number (MW285081). The associated BioProject and Bio-Sample numbers are PRJNA683709 (https://www.ncbi.nlm.nih.gov/bioproject/PRJNA683709) and SRX9654415 (https://www.ncbi.nlm.nih.gov/sra/SRX9654415/), respectively.

## References

[CIT0001] Aldrich J, Cherney BW, Merlin E, Christopherson L. 1988. The role of insertions/deletions in the evolution of the intergenic region between psbA and trnH in the chloroplast genome. Curr Genet. 14(2):137–146. 3180272 10.1007/BF00569337

[CIT0002] Ali A, Jaakko H, Poczai P. 2018. The chloroplast genome sequence of bittersweet (*Solanum dulcamara*): plastid genome structure evolution in Solanaceae. PLOS One. 13(4):e0196069. 29694416 10.1371/journal.pone.0196069 PMC5919006

[CIT0003] Huang K, Li ZR. 2013. Research on the ethnobotany of the nature vegetable *Zingiber* m*ioga* rose. Anhui Agric Sci Bullet. 19:55–57.

[CIT0004] Huang RS, Qian YL, Liu RN, et al. 2016. Ultrasonic-assisted extraction process of total flavonoids from *Zingiber mioga* and its antioxidant aactivity. Chin Pharm J. 51:1652–1656.

[CIT0005] Jo S-H, Cho C-Y, Lee J-Y, Ha K-S, Kwon Y-I, Apostolidis E. 2016. *In vitro* and *in vivo* reduction of post-prandial blood glucose levels by ethyl alcohol and water *Zingiber mioga* extracts through the inhibition of carbohydrate hydrolyzing enzymes. BMC Complement Altern Med. 16:111. 27036710 10.1186/s12906-016-1090-4 PMC4815155

[CIT0006] Kent WJ. 2002. BLAT-the BLAST-like alignment tool. Genome Res. 12(4):656–664. 11932250 10.1101/gr.229202 PMC187518

[CIT0007] Lee D-H, Ahn J, Jang YJ, Ha T-Y, Jung CH. 2016. *Zingiber mioga* reduces weight gain, insulin resistance and hepatic gluconeogenesis in diet-induced obese mice. Exp Ther Med. 12(1):369–376. 27347064 10.3892/etm.2016.3331 PMC4906615

[CIT0008] Lohse M, Drechsel O, Kahlau S, Bock R. 2013. OrganellarGenomeDRAW-a suite of tools for generating physical maps of plastid and mitochondrial genomes and visualizing expression data sets. Nucleic Acids Res. 41(Web Server issue):W575–W581. 23609545 10.1093/nar/gkt289 PMC3692101

[CIT0009] Luo R, Liu B, Xie Y, Li Z, Huang W, Yuan J, He G, Chen Y, Pan Q, Liu Y, et al. 2012. SOAPdenovo2: an empirically improved memory-efficient short-read de novo assembler. Gigascience. 1(1):18. 23587118 10.1186/2047-217X-1-18 PMC3626529

[CIT0010] Wang W, Yang T, Wang HL, et al. 2020. Comparative and phylogenetic analyses of the complete chloroplast genomes of six almond species (*Prunus* spp. L.). Sci Rep. 10:10137. 32576920 10.1038/s41598-020-67264-3 PMC7311419

[CIT0011] Wick RR, Schultz MB, Zobel J, Holt KE. 2015. Bandage: interactive visualization of de novo genome assemblies. Bioinformatics. 31(20):3350–3352. 26099265 10.1093/bioinformatics/btv383 PMC4595904

[CIT0012] Yi D-K, Choi K, Joo M, Yang JC, Mustafina FU, Han J-S, Son DC, Chang KS, Shin CH, Lee Y-M. 2016. The complete chloroplast genome sequence of *Abies nephrolepis* (Pinaceae: Abietoideae). J Asia Pac Biodivers. 9(2):245–249.

